# Sake lees hydrolysate protects against acetaminophen-induced hepatotoxicity via activation of the Nrf2 antioxidant pathway

**DOI:** 10.3164/jcbn.17-21

**Published:** 2017-09-05

**Authors:** Kayoko Kawakami, Chie Moritani, Misugi Uraji, Akiko Fujita, Koji Kawakami, Tadashi Hatanaka, Etsuko Suzaki, Seiji Tsuboi

**Affiliations:** 1 School of Pharmacy, Shujitsu University, 1-6-1 Nishigawara, Naka-ku, Okayama 703-8516, Japan; 2 Okayama Prefectural Technology Center for Agriculture, Forestry and Fisheries, Research Institute for Biological Sciences (RIBS), Okayama, 7549-1 Kibichuo-cho, Kaga-gun, Okayama 716-1241, Japan; 3 SATAKE Corporation, 2-30 Saijo Nishihonmachi, Higashi-Hiroshima-shi, Hiroshima 739-8602, Japan

**Keywords:** sake lees hydrolysate, acetaminophen, hepatotoxicity, Nrf2

## Abstract

Acetaminophen is a commonly used analgesic. However, an overdose of acetaminophen causes severe hepatotoxicity via depletion of hepatic glutathione. Here, we investigated the protective effects of sake lees hydrolysate against acetaminophen-induced hepatotoxicity in mice. Sake lees hydrolysate was administered orally to ICR mice for seven days. Six hours after acetaminophen treatment, the mice were sacrificed, and blood and liver samples were collected for analysis. Treatment with acetaminophen markedly increased the levels of serum alanine aminotransferase, aspartate aminotransferase, lactate dehydrogenase, and alkaline phosphatase. Pretreatment with sake lees hydrolysate significantly prevented the increases in the serum levels of these enzymes and inhibited acetaminophen-mediated glutathione depletion. In addition, histopathological evaluation of the livers also revealed that sake lees hydrolysate prevented acetaminophen-induced centrilobular necrosis. The expression of γ-glutamylcysteine synthetase (γ-GCS), hemeoxygenase-1 (HO-1) and nuclear factor erythroid 2-related factor 2 (Nrf2) in the liver were decreased after acetaminophen treatment, whereas pretreatment with sake lees hydrolysate led to an increased expression of all three proteins. Furthermore, sake lees hydrolysate induced the expression of these proteins in HepG2. These results suggested that sake lees hydrolysate could induces HO-1 and γ-GCS expression via activation of the Nrf2 antioxidant pathway, and protects against acetaminophen-induced hepatotoxicity in mice.

## Introduction

Acetoaminophen (APAP) is widely used as an analgesic and antipyretic drugs. APAP is metabolized by cytochrome P450 to form the highly reactive species, *N*-acetyl-*p*-benzoquinone imine (NAPQI), which under normal conditions is readily detoxified by conjugation with glutathione. However, an overdose of APAP causes an increase in the level of NAPQI, resulting in glutathione depletion. The binding of NAPQI to cellular macromolecules induces oxidative stress, ultimately leading to apoptosis and hepatic necrosis.^(1,2)^ Thus, inhibition of oxidative stress is considered to be an important strategy for treating APAP-induced hepatotoxicity.

Nuclear factor erythroid 2-related factor 2 (Nrf2) is a transcription factor that regulates the expression of antioxidant enzymes.^(3)^ Hemeoxygenase-1 (HO-1) and γ-glutamylcysteine synthetase (γ-GCS) are regarded as two of the most important antioxidant enzymes. We previously demonstrated that rice-derived peptides restored the expression of γ-GCS in mice with APAP-induced liver injury in addition to restoring glutathione levels.^(4)^ A previous study reported that induction of HO-1 expression contributes to protection against liver damage induced by APAP.^(5)^ Moreover, Nrf2-deficient mice have been reported to exhibit increased sensitivity to APAP.^(6)^ Therefore, Nrf2 has recently been implicated as a new therapeutic target for the treatment of liver disease.^(7)^

Sake lees are solid parts filtered from the mash of Japanese rice wine (sake) brewed with *Aspergillus oryzae* and *Saccharomyces cerevisiae*. Previous research demonstrated that sake lees hydrolysate (SLH) inhibits angiotensin I-converting enzyme activity.^(8)^ Sake lees serve as an attractive low-cost feedstock for the producion of functional food ingredient because they contain significant amounts of carbohydrate, protein, vitamins and other nutrients.^(9)^ However, most of these end up as industrial wastes due to limited demands. In addition, there has been no report on evaluating the effect of SLH against APAP-induced liver injury.

In this study, we evaluated the protective effect of SLH against APAP-induced liver injury in mice. We also investigated whether SLH regulates the expression of γ-GCS, HO-1, and Nrf2 in mice and HepG2 cells.

## Materials and Methods

### Materials

Sake lees were gifts from Muromachi Shuzo Co., Ltd. (Okayama, Japan). Denazyme AP, a protease from *Aspergillus oryzae*, was supplied by Nagase Chemtex Co., Ltd. (Osaka, Japan). Bathophenanthrolinedisulfonic acid, disodium salt (BAPS) was purchased from Dojindo Laboratories (Kumamoto, Japan). All other reagents were of the highest grade available.

### Preparation of SLH

SLH was prepared as previously described.^(10)^ In brief, 2 g of sake lees was dissolved in 40 ml of distilled water, adjusted to pH 7.5 with 5 N NaOH, and incubated with 1% (w/w) of Denazyme AP at 50°C for 17 h. The resultant hydrolysates were heated at 80°C for 30 min to inactivate the proteases. The hydrolysates were then centrifuged at 2,000 × *g* for 30 min. The supernatants were freeze-dried and stored at 4°C for further studies.

### Animals

Four-week-old male ICR mice were purchased from Japan SLC (Shizuoka, Japan). The animals were housed at 24 ± 1°C with a 12 h shift in the light/dark cycle and had free access to a standard diet and distilled water for 1 week prior to the experiment. All animal experimental protocols were approved by the Animal Experimentation Committee of Shujitsu University (Permit Number: 028-002); the study was conducted in accordance with the Animal Experimentation Guidelines of Shujitsu University.

Mice were randomly assigned to five groups. The SLH treatment groups were orally administered SLH (250 or 500 mg/kg body weight) daily for seven days with the use of a stomach tube. The control and APAP groups were administrated saline. All mice were fasted 18 h before intraperitioneal injection of APAP (700 mg/kg) and sacrificed 6 h after APAP treatment. Blood samples were collected and then centrifuged at 750 × *g* for 10 min at 4°C. The livers were collected from each mouse for Western blot and histopathological analysis.

### Cell culture

The human hepatoblastoma cell line HepG2 was obtained from RIKEN BRC Cell Bank (Ibaraki, Japan). The cells were cultured in Minimum Essential Medium (Sigma-Aldrich, St. Louis, MO) containing 10% fetal bovine serum, 25 mM HEPES (pH 7.4), 56 µg/ml amphotericin B, 100 U/ml penicillin, and 100 µg/ml streptomycin at 37°C in a humidified atmosphere of 5% CO_2_. A stock solution of SLH at a concentration of 1 g/ml was prepared in phosphate buffered saline. The cells were seeded at a density of 3 × 10^5^ cells/well in a 6-well plate (Thermo Fisher Scientific, Waltham, MA) and treated with 0–20 µl of SLH (a final concentrations of 0, 5, 10 and 20 mg/ml). Cells were harvested at 1, 3, 8, and 24 h for measurement of intracellular glutathione levels and Western blot analysis.

### Biochemical analysis

Serum alanine aminotransferase (ALT) and aspartate aminotransferase (AST) levels were measured using a Transaminase CII Test Wako kit (Wako Pure Chemical Industries Ltd., Osaka, Japan). The results are expressed in Karmen units. Lactate dehydrogenase (LDH) and alkaline phosphatase (ALP) were measured using Cytotoxicity Detection Kit^PLUS^ (Roche Applied Science, Mannheim, Germany) and LabAssay ALP (Wako Pure Chemical Industries Ltd.), respectively. Total glutathione (reduced and oxidized form) levels were measured using a previously described method.^(11)^ HepG2 cells and the livers were homogenized in 0.1 M HCl containing 1 mM BAPS. After deproteinization, the resulting supernatants were used to measure the total glutathione content.

### Histopathologic examination

Liver tissues were fixed in 10% phosphate buffered neutral formalin, dehydrated through a graded series of alcohol (50–100%) and embedded in paraffin. Thin sections were cut and stained with hematoxylin and eosin (H&E) stain for assessment by photomicroscopy.

### Western blotting analysis

For preparation of liver protein extracts, the livers were homogenized in RIPA buffer (Santa Cruz Biotechnology, Santa Cruz, CA). After incubation for 30 min at 4°C, the homogenates were centrifuged at 18,800 × *g* for 10 min at 4°C. The protein concentration of tissue homogenates was determined by the Bio-Rad protein assay (Bio-Rad, Hercules, CA). For immunoblot analysis, proteins were separated by sodium dodecyl sulfate-polyacrylamide gel electrophoresis (SDS-PAGE) and transferred electrophoretically to a polyvinylidene fluoride (PVDF) membrane. The membrane was blocked with PVDF Blocking Reagent (Toyobo, Osaka, Japan) for 1 h at room temperature. Immunoblotting was performed using primary antibodies against γ-GCS heavy subunit (γ-GCSh), γ-GCS light subunit (γ-GCSl), Nrf2 (Santa Cruz Biotechnology), HO-1 (Enzo Life Science, Farmingdale, NY), and β-actin (Sigma-Aldrich) for 1 h at room temperature. Bound antibodies were detected using secondary peroxidase-conjugated anti-rabbit or anti-mouse IgG (GE Healthcare, Buckinghamshire, United Kingdom). Target proteins were visualized using an ECL reaction solution (GE Healthcare).

### Statistical analysis

All data were expressed as the mean ± SEM. Statistical analysis was performed using ANOVA followed by the Dunnett’s test to determine significance between groups. *P* values lower than 0.05 were considered as statistically significant.

## Results

### Effect of SLH against APAP-induced liver injury in mice

**Fig. 1:**
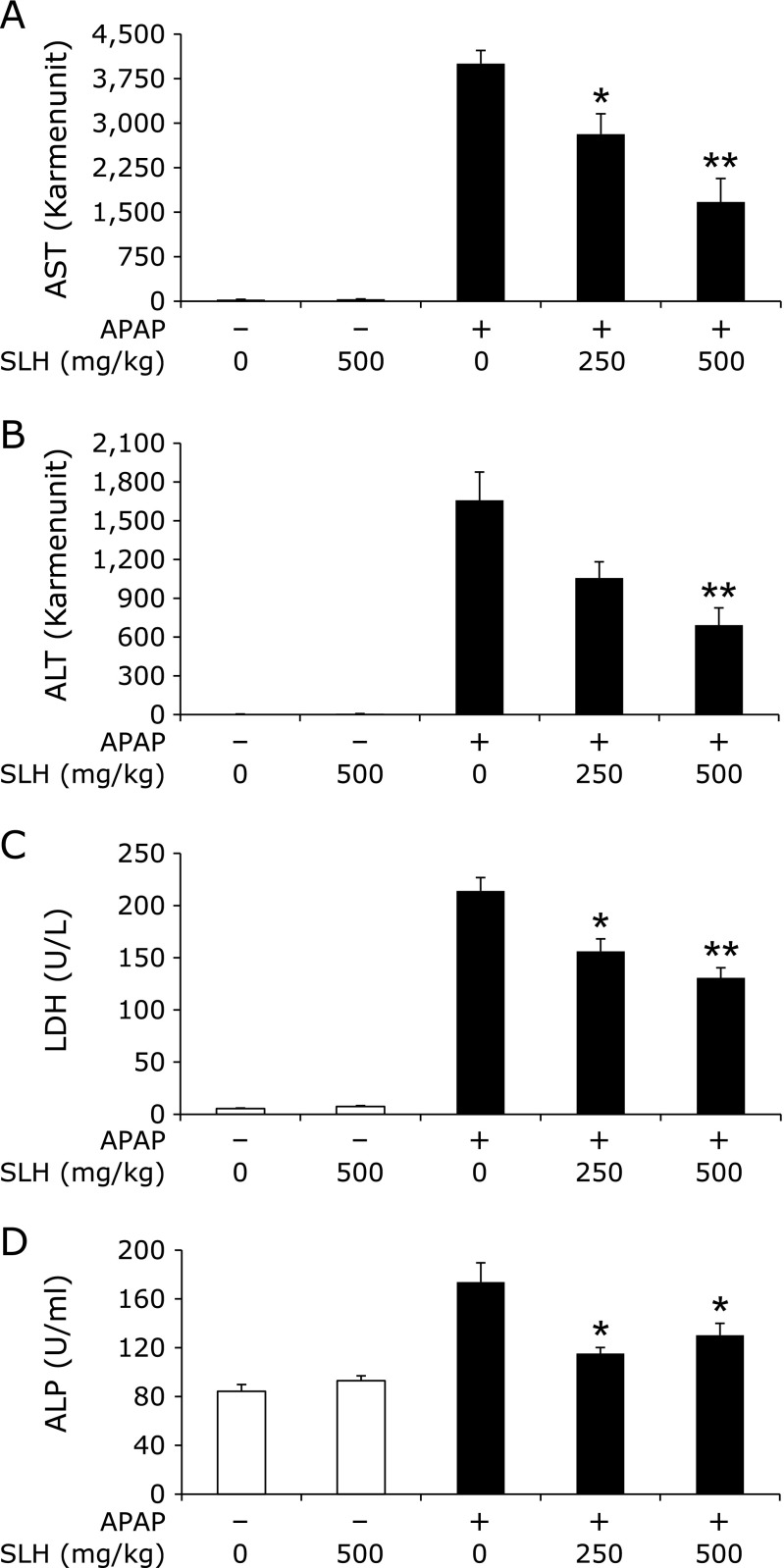
The effect of SLH on serum levels of AST (A), ALT (B), LDH (C) and ALP (D) in mice with liver injury induced by APAP. Values are mean ± SEM (*n* = 5–6). *p<0.05 and **p<0.01 vs APAP-treated group.

We assessed the protective effects of SLH on APAP-induced serum AST, ALT, LDH and ALP levels. No significant changes in body weight were observed among the groups throughout the 7-day treatment (Table 1). Administration of SLH alone did not affect the liver weight when compared to that of the control group, whereas the APAP-treated group showed a significant increase in liver weight compared to that of the control group. Pretreatment with SLH did not affect liver weight compared to that of the APAP-treated group. Basal serum levels of AST (Fig. 1A) and ALT (Fig. 1B) in the control group were 29.0 ± 2.1 and 5.0 ± 0.5 Karmen unit, respectively, and treatment with APAP for 6 h markedly increased AST and ALT levels to 4,003.5 ± 221.8 and 1,657.9 ± 219.2 Karmen unit, respectively. LDH (Fig. 1C) and ALP (Fig. 1D) levels were also elevated to 214.1 ± 12.7 U/L and 173.8 ± 15.8 U/ml, respectively, whereas the respective values for the control group were 5.4 ± 0.6 U/L and 84.3 ± 5.6 U/ml. Administration of SLH alone did not affect serum levels of these enzymes. In contrast, pretreatment with SLH significantly inhibited the elevation of serum AST, ALT, LDH and ALP levels.

**Fig. 2:**
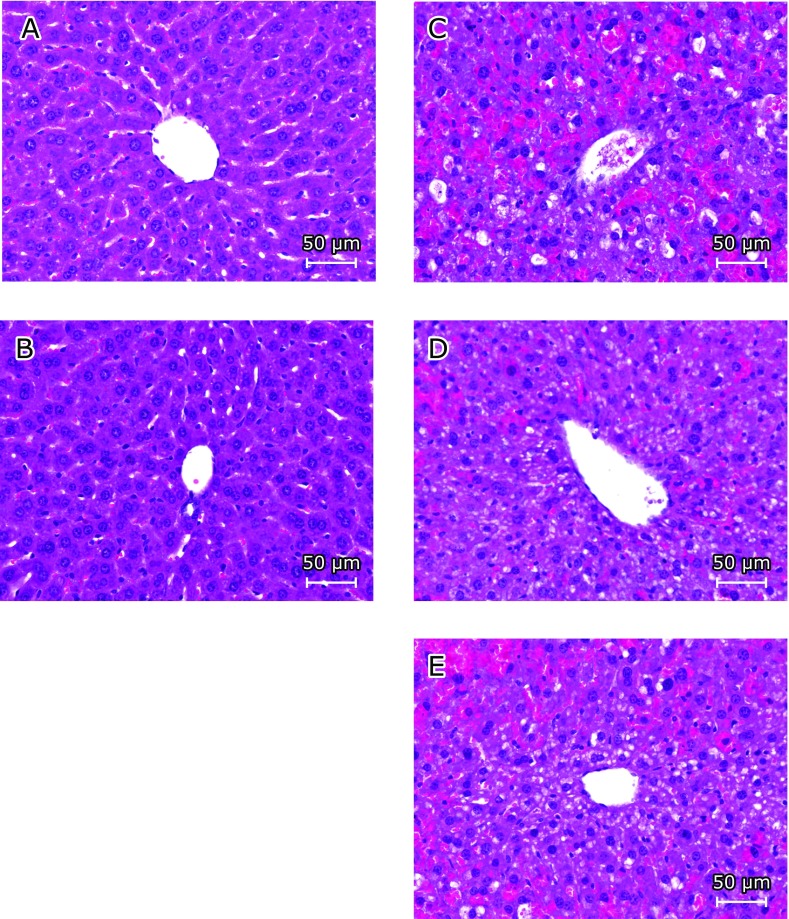
H&E staining of liver sections from APAP-treated mice. Representative images from each experimental group are shown (original magnification ×400). (A) Control group, (B) SLH 500 mg/kg only-treated group: normal lobular architecture and cell structure, (C) APAP-treated group: multiple and extensive areas of portal inflammation and hepatocellular necrosis, and a moderate increase in inflammatory cell infiltration, (D) SLH 250 mg/kg + APAP treatment group, and (E) SLH 500 mg/kg + APAP treatment group: mild portal inflammation and hepatocellular necrosis, and minimal inflammatory cell infiltration.

Glutathione, one of the most important antioxidant molecules, could scavenge NAPQI which is a toxic metabolite of APAP.^(12)^ Thus, we next measured the hepatic glutathione level. Pretreatment with SLH significantly recovered the APAP-induced glutathione depletion 6 h after APAP treatment (Table 1). Histological examination of the livers from the control (Fig. 2A) and SLH only-treated mice (Fig. 2B) showed normal lobular architecture and cell structure. APAP treatment induced multiple extensive areas of hepatic portal inflammation and hepatocellular necrosis, which were randomly distributed throughout the parenchyma, as well as a moderate increase in inflammatory cell infiltration (Fig. 2C), whereas SLH pretreatment ameliorated APAP-induced liver damage (Figs. 2D and E). These results indicate that SLH protects against APAP-induced liver injury.

### Effect of SLH treatment on γ-GCS, HO-1 and Nrf2 protein expression levels in the liver of APAP-treated mice

**Fig. 3:**
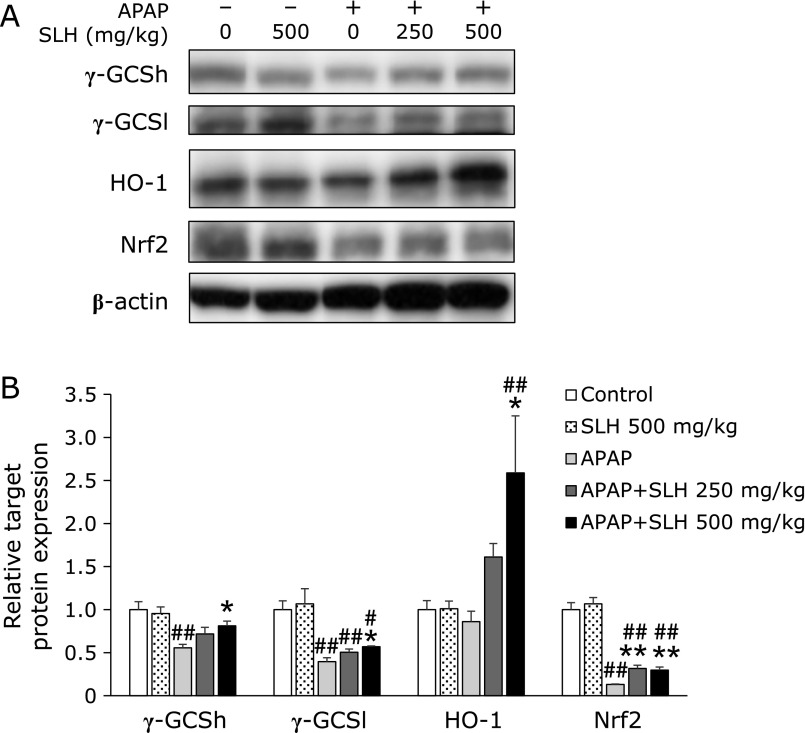
The expression levels of γ-GCS, HO-1 and Nrf2 in the liver of mice. Protein extracts from liver tissue were analyzed by SDS-PAGE and immunoblotting by using antibodies against γ-GCSh, γ-GCSl, HO-1 and Nrf2. (A) Western blot analysis of the expression of target proteins at 6 h after APAP treatment. Lane 1, Control; Lane 2, SLH 500 mg/kg; Lane 3, APAP; Lane 4, APAP + SLH 250 mg/kg; and Lane 5, APAP + SLH 500 mg/kg. (B) Densitometric analysis of Western blot. Values are mean ± SEM (*n* = 5–6). *p<0.05 and **p<0.01 vs APAP-treated group. ^#^*p*<0.05 and ^##^*p*<0.01 vs control group.

 HO-1 and γ-GCS, both of which are transcriptionally regulated by Nrf2, are important cellular antioxidant enzymes.^(13)^ Therefore, we observed whether the protective effect of SLH against APAP-induced liver injury is associated with Nrf2 and antioxidant enzymes. APAP treatment significantly decreased γ-GCSh, γ-GCSl and Nrf2 protein expression as compared to that in the control group (Fig. 3A, lane 3). No significant difference in HO-1 protein expression was observed between the APAP-treated and control groups. On the other hand, SLH dose-dependently increased the protein expression levels of γ-GCSh, γ-GCSl, HO-1 and Nrf2 (Fig. 3B). Pretreatment with SLH alone did not increase the expression levels of these proteins as compared to that reported for the control group (Fig. 3A, lane 2). These results suggest that the protective effect of SLH may be mediated through activation of the Nrf2 antioxidant pathway.

### Effect of SLH treatment on intracellular glutathione levels in HepG2 cells

**Fig. 4:**
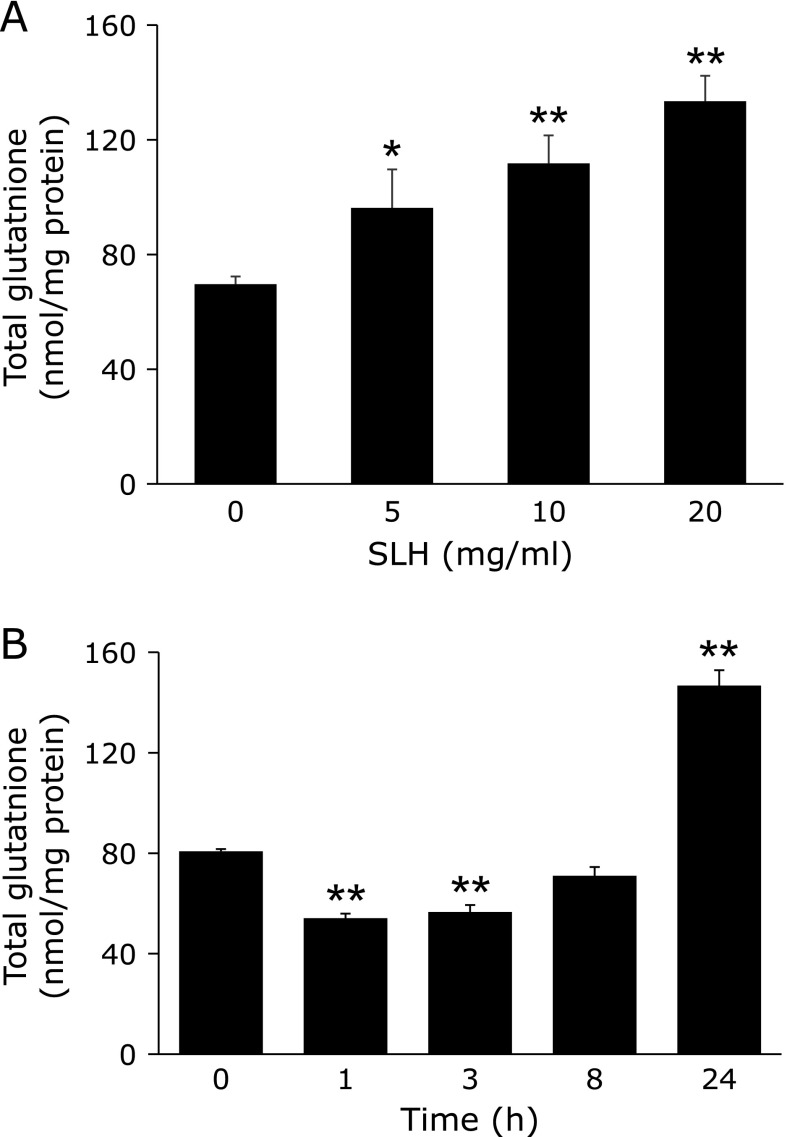
The effect of SLH on total glutathione levels in HepG2 cell. (A) Dose-dependent effect of SLH on total glutathione levels. Cells were treated with the indicated concentrations of SLH for 24 h. (B) Time-dependent effect of SLH on total glutathione levels. Cells were treated with SLH (20 mg/ml) for the indicated times. Values are mean ± SEM (*n* = 3). *p<0.05 and **p<0.01 vs control.

We next examined the effect of SLH on the levels of intracellular glutathione in HepG2 cells 24 h after treatment. SLH treatment increased intracellular glutathione levels in a dose-dependent manner (Fig. 4A). SLH treatment at 20 mg/ml elevated the level of glutathione to 113.4 ± 8.9 nmol/mg protein, which was 1.9-fold higher than the control that had a mean value of 69.6 ± 2.7 nmol/mg protein. Although SLH treatment at 20 mg/ml initially decreased glutathione levels at 1 and 3 h, the level was significantly increased at 24 h (Fig. 4B).

### Effect of SLH treatment on γ-GCS, HO-1 and Nrf2 protein expression in HepG2 cell

**Fig. 5:**
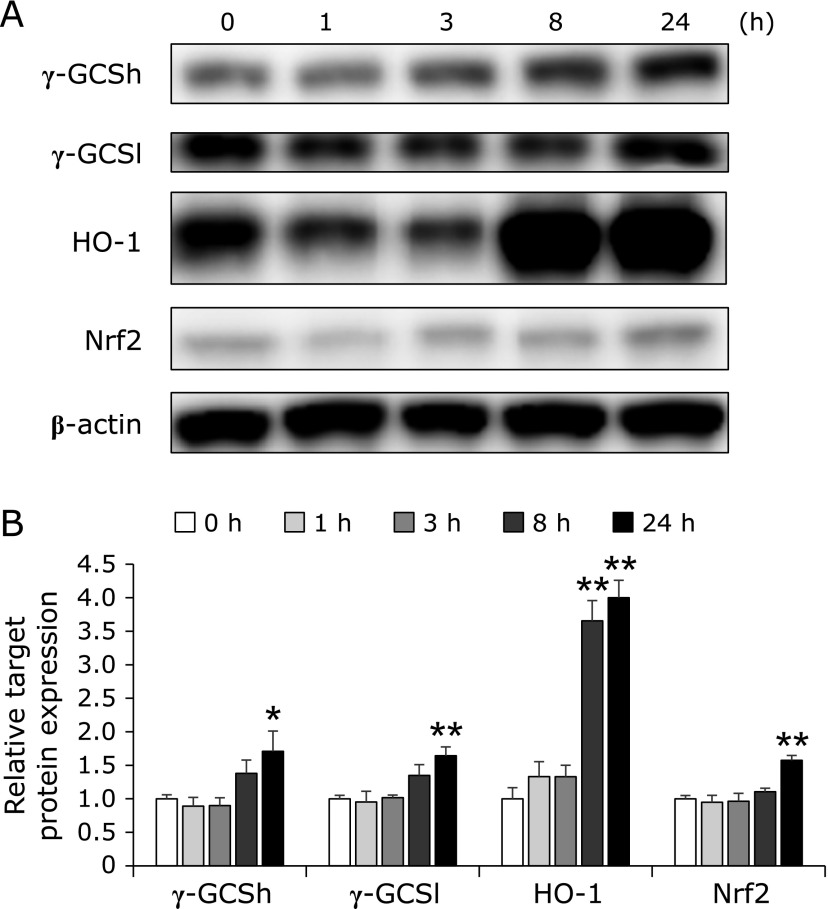
The expression levels of γ-GCS, HO-1 and Nrf2 in HepG2 cells. Protein extracts from HepG2 cells were analyzed by SDS-PAGE and immunoblotting by using antibodies against target proteins. (A) Western blot analysis of the expression of target proteins after SLH treatment at 20 mg/ml. (B) Densitometric analysis of Western blot. Values are mean ± SEM (*n* = 4). *p<0.05 and **p<0.01 vs 0 h.

We further examined the protein expression levels of γ-GCSh, γ-GCSl, HO-1 and Nrf2 after SLH treatment in HepG2 cells. HepG2 cells were treated with SLH at 20 mg/ml at the indicated time points. The time-course profile of γ-GCSh and γ-GCSl correlated with that of the glutathione levels. SLH treatment significantly increased γ-GCSh, γ-GCSl, HO-1, and Nrf2 protein expression at 24 h (Fig. 5A). In particular, HO-1 protein expression was induced by 4.0-fold 24 h after SLH treatment (Fig. 5B).

## Discussion

We assessed the effect of oral administration of SLH on APAP-induced liver injury. The serum AST, ALT, LDH and ALP levels are the most sensitive biomarkers of hepatic damage. APAP caused a significant increase in the levels of AST, ALT, LDH and ALP (Fig. 1), however, pretreatment with SLH significantly decreased the serum levels of these enzymes. Moreover, results from histological observations of the H&E stained liver sections showed that SLH significantly decreased hepatic necrosis (Fig. 2). In this study, we found that SLH protects liver tissue against APAP-induced damage.

NAPQI, a toxic metabolite of APAP, is detoxified by glutathione to form APAP-glutathione adducts.^(14)^ Due to the important role played by glutathione in the antioxidant defense system, it may serve as a key determinant of APAP-induced hepatotoxicity. Therefore, we measured hepatic glutathione levels. Pretreatment with SLH significantly restored glutathione levels that were reduced by APAP treatment, while administration of SLH alone did not affect hepatic glutathione levels compared to the control (Table 1). Glutathione is an abundant cellular antioxidant and is maintained at a stable level in cells.^(15)^ These results suggest that SLH may accelerate hepatic glutathione recovery. The enzyme γ-GCS catalyzes the first and rate-limiting step in glutathione biosynthesis.^(16,17)^ A previous study showed that rice-derived peptides protect against APAP-induced liver injury by up-regulating γ-GCS expression.^(4)^ In this study, we demonstrated that SLH induced γ-GCS expression in the liver of mice (Fig. 3B). These results suggest that SLH may accelerate hepatic glutathione recovery.

It is generally known that γ-GCS and HO-1 are antioxidant enzymes. HO-1 is responsible for the catabolism of heme release from drug metabolizing enzymes to protect against oxidant-induced tissue injury.^(5)^ Previous studies have shown that sulforaphane plays a protective role against APAP-induced hepatotoxicity though antioxidant effects mediated by HO-1 induction.^(18)^ In this study, we demonstrated that SLH induced HO-1 expression in the liver of mice (Fig. 3B). In particular, SLH (500 mg/kg) increased the protein expression level of HO-1 by 2.6-fold in the liver of APAP-treated mice. Induction of γ-GCS and HO-1 is mainly due to transcriptional activation mediated by Nrf2.^(13,19)^ Nrf2 is an important transcription factor, which has been reported to play a key protective role against APAP-induced liver injury.^(20)^ Our results showed that SLH reversed the APAP-induced decrease in Nrf2 expression (Fig. 3B). We also demonstrated that intracellular glutathione levels were significantly increased upon treatment with SLH in HepG2 cells (Fig. 4A). In addition, the increase in glutathione levels correlated with increased expression of γ-GCS and Nrf2 at 24 h (Fig. 4B and 5B). Previous reports have demonstrated that some natural products, such as caffeic acid,^(21)^ esculentoside A,^(22)^ salvianolic acid B,^(23)^ quercetin^(24)^ and sauchinone,^(25)^ protect against APAP-induced liver injury by enhancing Nrf2 transcription. Our results indicate that SLH activates Nrf2, which then enhances the expression of γ-GCS and HO-1, and contributes to the protective effect of SLH against APAP-induced liver injury. Posttranscriptional modifications of Nrf2 by kinases such as protein kinase C, phosphatidylinositol 3-kinase, mitogen-activated protein kinase, and glycogen synthase kinase-3β, have been associated with Nrf2 activation.^(26–29)^ Further studies are required to evaluate the mechanisms involved in SLH-induced Nrf2 activation.

The protein content in sake lees is as high as 44.6%, along with 37.4% carbohydrate, 6.7% fiber, 2.5% fat and 1.8% ash.^(9)^ Therefore SLH contains various peptides, which are generated sake lees proteins by hydrolysis using Denazyme AP, containing mainly exopeptidases. Our previous study showed that Denazyme AP generated Pro-containing di- and tripeptides.^(10)^ Thus, active ingredients of SLH are expected to be Pro-containing di- and tripeptides. Di- and tripeptides are directly absorbed into the body via the peptide transporter PEPT1 on the intestinal brush border membrane.^(30,31)^ For instance, orally administered Val-Pro-Pro and Gly-Pro-Hyp remain intact form in the intestine,^(32,33)^ retaining their activity until adsorption. Accordingly, SLH could be resistant to digestion and exert their physiological effects in the body.

Protein hydrolysates from rice, rice bran, eggshell membrane and chickpea have been reported to increase the intracellular glutathione levels and upregulate anti-oxidative enzymes in cells.^(4,34–36)^ Since the active concentration ranged from 0.1 to 10 mg/ml, SLH showed similar bioactivity to the aforementioned hydrolysates (Fig. 4A).

SLH induced the expression of HO-1 at 8 and 24 h, this induction preceded the induction of Nrf2. The transcription factors activator protein (AP)-1 and nuclear factor-κB (NF-κB) have been implicated in the induction of HO-1.^(37,38)^ Thus, induction of HO-1 by SLH also may be mediated via AP-1 or NF-κB activation.

In conclusion, our results indicate that SLH has a potent hepatoprotective effect against APAP-induced liver injury in mice. Our findings suggest that SLH could play important roles in the prevention of oxidative stress-associated disease though regulation of the Nrf2 pathway.

## Figures and Tables

**Table 1:** The effect of SLH on body weight, liver weight and hepatic glutathione levels in mice with liver injury induced by APAP

Groups	Body weight (g)	Liver weight (mg/g body weight)	Total glutathione (µmol/g liver)
control	29.8 ± 1.7	39.9 ± 0.9	3.8 ± 0.3
SLH 500 mg/kg	29.8 ± 1.8	43.4 ± 0.7	3.5 ± 0.6
APAP	30.5 ± 0.3	71.8 ± 1.8^#^	0.012 ± 0.001^#^
APAP + SLH 250 mg/kg	29.7 ± 0.3	70.9 ± 1.9^#^	0.047 ± 0.008*^,#^
APAP + SLH 500 mg/kg	29.9 ± 0.9	68.9 ± 2.4^#^	0.040 ± 0.009*^,#^

Values are mean ± SEM (*n* = 5–6). *p<0.05 vs APAP-treated group. ^#^*p*<0.01 vs control group.
